# Creation and validation of a ligation-independent cloning (LIC) retroviral vector for stable gene transduction in mammalian cells

**DOI:** 10.1186/1472-6750-12-3

**Published:** 2012-01-16

**Authors:** Asmita Patel, Anisleidys Muñoz, Katherine Halvorsen, Priyamvada Rai

**Affiliations:** 1 Department of Medicine, University of Miami Miller School of Medicine, 1600 N.W. 10th Avenue, Miami, FL 33136, USA; 2 Biology Department, Cox Science Center, 1301 Memorial Drive, University of Miami, Coral Gables, FL 33124, USA; 3 Sylvester Comprehensive Cancer Center, University of Miami Miller School of Medicine, Miami, 1475 N.W. 12th Avenue, FL 33136, USA

## Abstract

**Background:**

Cloning vectors capable of retroviral transduction have enabled stable gene overexpression in numerous mitotic cell lines. However, the relatively small number of feasible restriction enzyme sequences in their cloning sites can hinder successful generation of overexpression constructs if these sequences are also present in the target cDNA insert.

**Results:**

Utilizing ligation-independent cloning (LIC) technology, we have modified the highly efficient retroviral transduction vector, pBABE, to eliminate reliance on restriction enzymes for cloning. Instead, the modified plasmid, pBLIC, utilizes random 12/13-base overhangs generated by T4 DNA polymerase 3' exonuclease activity. PCR-based introduction of the complementary sequence into any cDNA of interest enables universal cloning into pBLIC. Here we describe creation of the pBLIC plasmid, and demonstrate successful cloning and protein overexpression from three different cDNAs, Bax, catalase, and p53 through transduction into the human prostate cancer cell line, LNCaP or the human lung cancer line, H358.

**Conclusions:**

Our results show that pBLIC vector retains the high transduction efficiency of the original pBABE while eliminating the requirement for checking individual cDNA inserts for internal restriction sites. Thus it comprises an effective retroviral cloning system for laboratory-scale stable gene overexpression or for high-throughput applications such as creation of retroviral cDNA libraries. To our knowledge, pBLIC is the first LIC vector for retroviral transduction-mediated stable gene expression in mammalian cells.

## Background

Cloning vectors capable of being packaged into retroviral particles for transduction into mammalian cells provide efficient tools for stably altering the genome of dividing cells. In this regard, the Moloney murine leukemia virus (MMLV)-based pBABE vector system [1] has been widely utilized for highly efficient stable gene overexpression in a variety of different mammalian cells with negligible off-target cellular effects. The pBABE vector consists of a bacterial origin of replication, viral elements for gene packaging, transcription and processing, and a unique restriction enzyme sequence-based cloning site http://www.addgene.org/1767/. Additionally it contains an ampicillin-resistance gene for selection in bacteria, and either puromycin, hygromycin or neomycin (G418)- resistance genes in order to select for stably transduced cell lines [1]. This is the first retroviral transduction system that does not require helper viruses. Instead either amphotropic or ecotropic viral env gene-expressing and gag-pol gene-expressing plasmids are co-transfected along with the desired pBABE construct into an appropriate packaging cell line to produce high-titer, replication-incompetent viruses for the transfer and expression of exogenous genes in mammalian cells [1]. The ability to use pBABE constructs as part of a three-plasmid packaging system greatly reduces the probability of recombination events leading to horizontal transfer and live virus production in the transduced lines, making it a safe and effective retroviral gene transduction system.

A major disadvantage of the pBABE plasmid is its relatively limited cloning site http://www.addgene.org/1767/. To increase cloning efficiency, two different enzymes need to be selected from the cloning site so as to generate non-complementary overhangs in the digested DNA, thus preventing self-ligation. When such a double digestion protocol is used, a series of conditions must be taken into consideration. Besides the absence of each enzyme site in the target DNA, the two enzymes should share a common set of optimal reaction conditions, including salt concentrations, digestion times and activation/inactivation temperatures. Lack of such compatible digestion conditions necessitates tedious and time-consuming sequential digestions as well as loss of DNA product during intermediate purifications. The enzymes that best fit these criteria in the pBABE multiple cloning site are SalI, BamHI and EcoRI. In our experience [2,3], using SalI does not uniformly yield optimal digestion in combination with either BamHI or EcoRI, leading to reduced cloning efficiency. Additionally if any of these three sites, particularly BamHI or EcoRI, are internally present within the target cDNA, then the cloning process becomes extremely complicated.

Ligation-independent cloning (LIC) technology was developed to remove the conventional requirements of compatible restriction enzyme sites and exogenous enzymatic ligation during the cloning process [4]. This methodology has been used for PCR-based amplification of genomic DNA sequences [4], to generate vectors for bacterial protein overexpression [5], for high-throughput cloning of biocatalysts from prokaryotic genomes [6], for the addition of variable-length C-terminal histidine tags [7], and for gene silencing in plant cells [8]. However to our knowledge, there are no LIC retroviral vectors available for stably overexpressing proteins in mammalian cells.

Therefore, to increase cloning applicability and efficiency, we adapted the pBABE retroviral plasmid backbone into a LIC version denoted as pBLIC (Additional File 1, Figure S1). Unlike pBABE, cloning into this vector no longer requires conventional restriction site-generated sticky ends but instead relies on a random 12/13-sequence comprised of 3 bases. The 12/13-mer sequences are designed such that overhangs are generated using the 3' exonuclease activity of T4 polymerase. The complementary overhang sequence can be added to target cDNA inserts via PCR extension from a primer template. Because the overhang is generated by exonuclease rather than endonuclease activity and requires a free 3' end, it will not be processed even if it does happen to be internally present in the target cDNA.

Here we outline the strategy we followed to modify the pBABE plasmid vector into pBLIC. We demonstrate successful cloning of three different cDNA for proteins of different molecular weights, Bax (MW: 26 kDa), catalase (MW: 60 kDa) and p53 (MW: 53 kDa) into the adapted vector, and protein overexpression from the transduced constructs in the LNCaP human prostate cancer cell line. Thus our results support wide applicability of the modified pBLIC plasmid for mammalian retroviral transduction and protein overexpression without consideration of restriction enzymes sites or prior ligation during the cloning process. The rationale behind our basic protocol can be readily used to adapt other retroviral plasmids. Thus our method provides an easy and rapid universal method of introducing any DNA sequence of interest into the modified LIC retroviral vector, enhancing the efficiency of laboratory-scale cloning. Further the LIC retroviral system can greatly facilitate high-throughput cloning processes such as those used for the creation and verification of retroviral gene overexpression libraries where screening individual inserts for internal restriction enzyme sites is impractical.

## Results and Discussion

**Figure 1:**
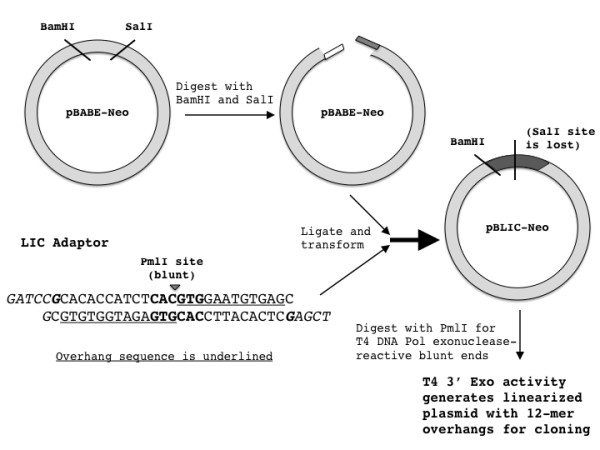
**Schematic depicting the conversion of the pBABE-Neo plasmid into pBLIC-Neo by insertion of a specially designed LIC adaptor**. The LIC adaptor consists of a PmlI site and a 12/13 base pair sequence used for LIC cloning (for generating overhangs consisting of 13 bases on the left side and 12 bases on the right). The PmlI restriction sequence is bolded and the digestion site is indicated by an inverted triangle within the restriction sequence. The terminal overhang sequences for BamHI (right) and SalI (left) that allow ligation of the LIC adaptor into the original pBABE-Neo plasmid are italicized. Note that the palindromic nature of the SalI site (G/TCGAC) will be lost upon ligation of the adaptor sequence with the digested pBabe plasmid (CTCGAC). The guanines that serve as T4 exonuclease activity termination sites are bolded and italicized.

The goal of our study was to convert the pBABE retroviral vector into an LIC version in order to remove the requirement for screening target cDNA inserts for absence of desired cloning restriction enzyme sites and for more universally efficient cloning. Eliminating restriction enzymes from the cloning process necessitates utilizing another means of generating complementary DNA overhangs in the plasmid and target insert. This was achieved by using the 3' exonuclease activity of T4 DNA polymerase and site-specifically inhibiting the competing 5' polymerase activity by leaving out a subset of deoxynucleotides from the reaction mixture, a technique that has been previously used to generate LIC vectors [4]. The adaptor module added to convert pBABE into pBLIC (Figure 1) was designed to facilitate this manner of overhang generation. Therefore it involved three elements: the addition of a blunt cutter enzyme site not present in the original plasmid, restriction enzyme sites corresponding to the cloning site in pBABE to allow ligation of the adaptor insert, and 12/13-mer sequences consisting of three randomly repeated bases (excluding the base corresponding to the added deoxynucleotide) that would constitute the overhang.

**Figure 2:**
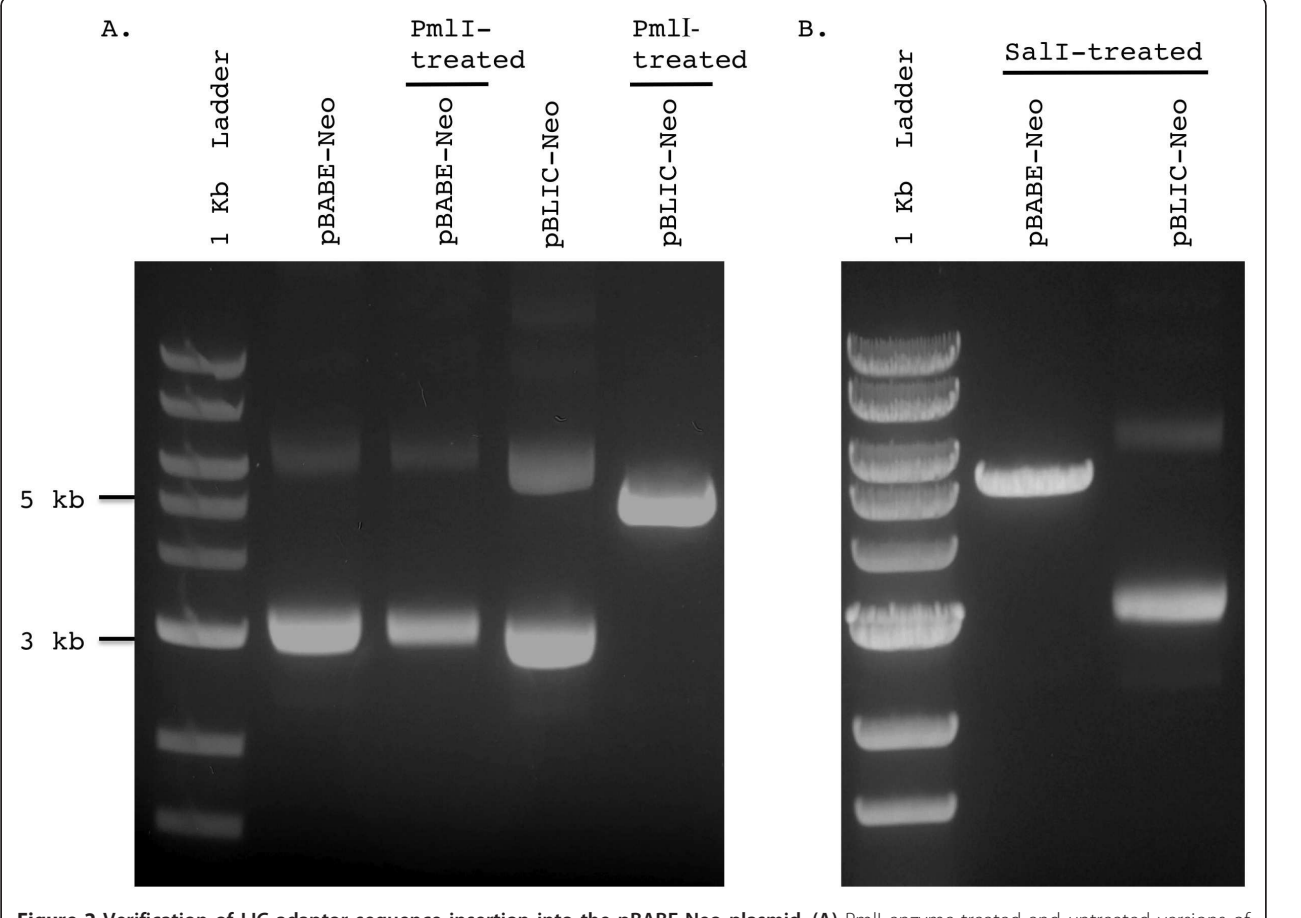
**Verification of LIC adaptor sequence insertion into the pBABE-Neo plasmid**. **(A) **PmlI enzyme-treated and untreated versions of both pBABE and pBLIC plasmids were run on a 0.7% agarose gel to check for insertion of the adaptor via presence of a PmlI site. A 1 kb ladder (NEB, N3232S) is used to mark band sizes. Gels were photographed on a Canon Powershot G10 mounted on a UVP Multidoc-it Digital Imaging System using standard preset contrast settings. Note that for the digested pBLIC, a single band corresponding to the linearized plasmid (~ 5 kb) is observed whereas the treated pBABE plasmid yields the same set of bands as the undigested plasmid. **(B) **Both pBABE and pBLIC were digested with SalI to check for loss of its restriction site, as expected upon successful insertion of the LIC adaptor sequence. For pBABE, a single band corresponding to a linearized plasmid at ~ 5 kb is seen whereas for pBLIC, the super-coiled and nicked forms are observed, indicating lack of digestion.

The first step involved identifying a blunt cutter enzyme not present in the pBABE plasmid. Accordingly, we added a PmlI consensus sequence into the adaptor insert (Figure 1). Thus once the adaptor was successfully inserted into the pBABE backbone, digestion with PmlI yielded the linearized plasmid for the pBLIC version but not for the original pBABE version (Figure 2A). The linearized form acts as a template for T4 exonuclease activity.

For the sequence overhangs generated upstream of the PmlI digestion site (Figure 1), we used a random combination of A, T and C such that it was unlikely to correspond to a restriction enzyme site or coding DNA. The overhang was maintained by leaving out dCTP, dATP and dTTP from the reaction mixture, thus inhibiting T4 5' DNA polymerase activity from filling in the overhang. Conversely, digestion past the desired overhang sequence (Figure 1) was prevented by addition of dGTP to the reaction mixture, allowing polymerase activity to proceed as soon as the bold italicized G in the adaptor sequence is encountered (Figure 1). The choice of which three bases to be used to generate the random overhang is dependent on the three bases 5' of digestion site in the restriction sequence of the single cutter enzyme. For instance the restriction sequence for PmlI is CAC-GTG, where the hyphen denotes the digestion site that leads to the blunt 3' end. Thus C and A were necessarily included in the pBLIC adaptor sequence (Figure 1).

Finally we added SalI and BamHI overhang sequences to the ends of the adaptor module (Figure 1). Note that only the overhang sequences that are generated via restriction digestion (as opposed to the full palindromic consensus sequence) were added. The pBABE plasmid was digested with these two enzymes in order to remove its original cloning site and to permit ligation of the adaptor insert into the plasmid to generate pBLIC. This process restored the full BamHI site but the palindromic nature of the SalI site was destroyed (Figure 1).

Conversion of the pBABE plasmid into pBLIC was also confirmed by digestion with PmlI. Subsequent to PmlI digestion, as expected in the pBABE plasmid, the gel exhibited bands corresponding to the fully intact, nicked and linearized forms of the plasmids (Figure 2A). In contrast, the vector with the ligated LIC insert showed a single band corresponding to a linearized ~5 kb plasmid (Figure 2A). Ligation of the LIC module into the digested pBABE plasmid destroys the SalI unique cloning site present in the original plasmid. Accordingly, when both pBABE and pBLIC were digested with SalI, pBLIC no longer yielded the linearized band (Figure 2B). Finally insertion of the LIC adaptor was verified by sequencing with the SV40 promoter reverse primer 5' GAAATTTGTGATGCTATTGC 3'.

**Figure 3:**
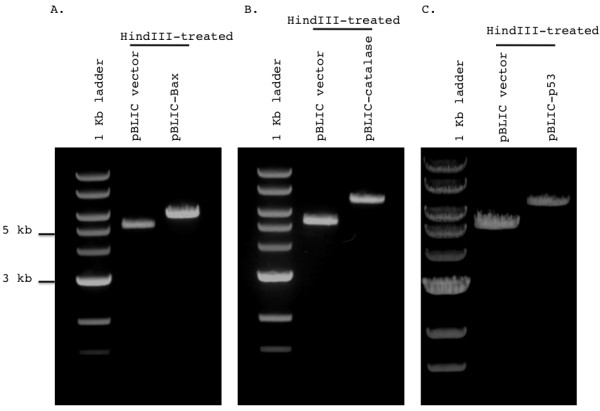
**Verification of Bax, catalase or p53 cDNA insertion into pBLIC**. Clones were digested with HindIII and run on a 0.7% agarose gel. A 1 kb ladder is used to mark band sizes. Gels were photographed on a Canon Powershot G10 mounted on a UVP Multidoc-it Digital Imaging System using standard preset contrast settings. The mobility shift relative to the empty pBLIC vector indicates presence of the cDNA insert. Restriction digest products for **(A) **pBLIC-Bax, **(B) **pBLIC-catalase, and **(C) **pBLIC-p53 are shown. Note that the shift in HindIII-treated plasmids is greater for catalase and p53 relative to Bax, corresponding to the larger insert size of the former two cDNAs relative to the latter (catalase: 1584 bp, p53: 1182 bp vs. Bax: 657 bp).

Once we verified conversion of pBABE to the pBLIC version, we wanted to validate its ability to be used as a gene overexpression vector that can be virally packaged and transduced in mammalian cells. To do so, we used the pBLIC vector to clone three cDNA constructs that are of interest to our laboratory, the pro-apoptotic protein Bax, the antioxidant protein, catalase and the tumor suppressor/DNA damage-responsive protein, p53. These had previously proved difficult to clone into the pBABE vector due to the presence of internal BamHI and/or EcoRI sites in their cDNA sequences. As described in the Methods section and outlined above, PCR primers were designed to add 12/13-mer sequences complementary to the T4-generated pBLIC overhangs (Figure 1) to the cDNA to allow ligation of the plasmid and insert. This was accomplished by PCR elongation from the appropriate primer template (Table 1). The resulting duplex segment was treated with T4 polymerase, with only dCTP added to the reaction mixture, in order to produce complementary overhangs. The co-mixed insert and plasmid (as described in Methods) were used to transform XL-10 gold recombination-deficient E. coli, and ligation was accomplished endogenously by the bacterial DNA repair enzymatic machinery. The resulting constructs were purified from bacterial pellets and were verified both by internal digestion at a unique HindIII site to yield a fragment of lower mobility containing the insert (Figure 3A-C) relative to the empty vector as well as by DNA sequencing (Additional File 1, Figure S2).

**Table 1:** PCR primers for cloning into pBLIC

cDNA	Primer Sequence
Bax	Fwd: 5' **CACACCATCTCAC*G***GCCACCATGGACGGGTCCGGGGAGCAG 3'
	Rev: 5' **CTCACATTCCAC*G***TCAGCCCATCTTCTTCCAGATG 3'

Catalase	Fwd: 5' **CACACCATCTCAC*G***GCCACCATGGCTGACAGCCGGG 3'
	Rev: 5' **CTCACATTCCAC*G***GGTGGCTCACAGATTTGCCTTCTC 3'

p53	Fwd: 5' **CACACCATCTCAC*G***GCCACCATGGAGGAGCCGCAGTCAGATCC 3'
	Rev: 5' **CTCACATTCCAC*G***TCAGTCTGAGTCAGGCCCTTCTGT 3'

Overhang sequences are **bolded**, the T4 exonuclease activity termination site guanine is ***bolded and italicized***, and the Kozak sequence is underlined.

**Figure 4:**
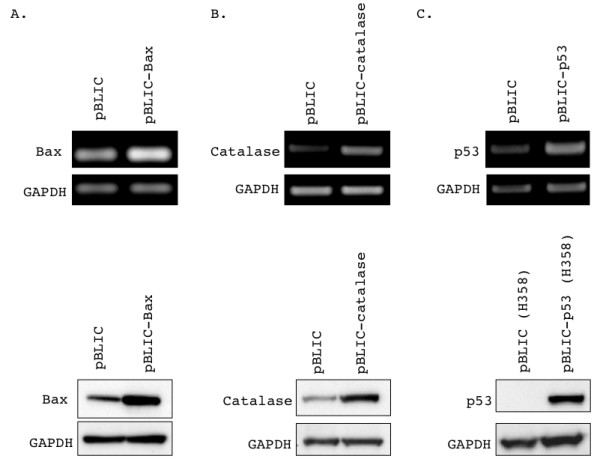
**Verification of pBLIC-Bax, pBLIC-catalase or pBLIC-p53 transduction and protein overexpression**. Either LNCaP or H358 cells were transduced with the indicated overexpression constructs and selected in G418-containing media as described in the Materials and Methods section, and harvested within 7 days of selection. RT-PCR and Western blot analysis was used to confirm overexpression of mRNA and protein from the constructs. RT-PCR and Western blotting was carried out on RNA and protein samples from cells transduced with the indicated constructs. Cells transduced with the empty pBLIC-Neo vector were used to determine baseline levels of the two proteins. Single bands were seen corresponding to the full-length mRNA (top panel, RT-PRCR) or protein (bottom panel, Western blotting) in all cases. GAPDH is shown as a loading control for both RT-PCR and Western blotting. **(A) **Overexpression of Bax mRNA and protein from pBLIC-Bax in LNCaP cells. **(B) **Overexpression of catalase mRNA and protein from pBLIC-catalase in LNCaP cells. **(C) **Overexpression of p53 mRNA in LNCaP cells and p53 protein in H358 cells from the pBLIC-p53 construct.

The purified pBLIC cDNA constructs were used to transduce the human prostate cancer cell line LNCaP, as described in Methods. The modified construct appeared to be as efficiently packaged into viral particles as the parental pBABE plasmid judging by the lysed appearance of the packaging HEK 293T cells (not shown). The transduced cells were continuously selected in G418-containing media. Mock-transduced LNCaP cells were used as a "canary" plate - all cells in this plate died within a week whereas approximately 20% cells died in the transduced plates within the same time period. Subsequently cells showed minimal death in G418-containing media, indicating that only the successfully transduced cells had survived. Cells harvested from the empty pBLIC-neo and pBLIC-Bax, pBLIC-catalase or pBLIC-p53 transduced samples were subjected to RT-PCR and Western blotting to determine expression of the target cDNA. Both Bax (Figure 4A) and catalase (Figure 4B) constructs showed robust overexpression of the respective full-length mRNA and protein in the transduced LNCaP cells. However while the pBLIC-p53 construct yielded p53 mRNA overexpression in LNCaP cells (Figure 4C), we were unable to detect significant p53 protein overexpression in these cells (not shown). Because the transcript was overexpressed and the LNCaP cells exhibited G418 resistance, we surmised that the excess p53 was being post-translationally targeted for degradation by Mdm2 in the LNCaP cells, which possess a fully functional p53 pathway [9]. Accordingly we also transduced the pBLIC-p53 construct in the p53-null [10] H358 lung cancer cell line. In this background, we were able to detect a high degree of p53 protein overexpression from pBLIC-p53 (Figure 4C).

## Conclusions

In this study, we demonstrate successful conversion of the pBABE retroviral transduction vector into an LIC version, pBLIC. The pBLIC vector retains the highly efficient transduction and overexpression capability of the original pBABE with the added advantages of ligation-independent cloning. Although we present here the results demonstrating gene overexpression using the converted pBABE plasmid with neomycin (G418) resistance, we have also converted the pBABE-puromycin plasmid by the same process (not shown). Furthermore the general strategy outlined above can be easily utilized to convert other retroviral plasmids such as pWZL, pLXSN or pMIG into LIC versions. Given the popularity and efficiency of the pBABE vector system for retroviral transduction and overexpression of cDNAs, we believe that the pBLIC version will simplify and extend its applications, for instance by making it a feasible vector for high-throughput cloning applications such as generation of cDNA libraries.

## Methods

### Cell culture

LNCaP cells were cultured in RPMI-1640 medium containing 5% fetal bovine serum and 100 units/ml penicillin-streptomycin. H358 cells were cultured in RPMI-1640 in 10% fetal bovine serum and 100 units/ml penicillin-streptomycin. HEK-293T cells were cultured in Dulbecco's Modified Eagle Medium (DMEM) containing 10% fetal bovine serum and 100 units/ml penicillin-streptomycin. All cell culture reagents were obtained from Gibco, Invitrogen.

### Design of the LIC adaptor segment

The LIC segment was designed to contain SalI and BamHI overhangs to allow ligation into the pBABE cloning site. It also contained the following 12/13-mer sequences to create overhangs via T4 exonuclease activity (13 base overhang on the left below, 12 base overhang on the right):

**5' GATCC*G***CACACCATCTCAC*GTG**GAATGTGAG*C 3'

3' GC*GTGTGGTAGA**GT*GCACCTTACACTC***G*AGCT 5'**

The **bolded **letters indicate the complementary SalI and BamHI sites used to ligate the segment into the pBABE backbone. The underlined site is for the unique blunt cutter PmlI to generate the ends/starting site for 3' T4 polymerase exonuclease activity. The 12/13-base sticky ends generated by this activity are *italicized*. The ***bold, italicized ***Gs are stopping points for T4 exonuclease activity. This is accomplished by adding only dGTP to the reaction mixture. Thus the chemical equilibrium for T4 polymerase shifts from the 3'-> 5' exonuclease activity to the 5'-> 3' polymerase activity only once the italicized bolded G is encountered by T4 polymerase as the other complementary nucleotides to the italicized overhang are absent. The above oligonucleotides (Integrated DNA Technologies) were used to generate the adaptor segment by annealing in a 1:1 ratio (5 μl of 1 μg/μl each in NEB Buffer 2).

### Construction and preparation of the modified backbone vector

The pBABE-neomycin vector was digested with BamHI and SalI to remove the original cloning site and the resulting segment was gel purified using the Qiagen gel purification kit according to manufacturer instructions. The annealed LIC adaptor segment (described above) was diluted 1:10 and mixed with 50 ng of the purified digested pBABE vector prior to ligation using a Rapid DNA ligation kit (Roche) as per manufacturer's instructions. The resulting pBLIC plasmid was digested with PmlI to generate the linearized plasmid. This was subsequently treated with T4 polymerase (NEB). Treatment conditions were as follows: 0.4 pmol digested vector, 2 μl 10X Buffer 2 (NEB), 2 μl dGTP (Roche), 2 μl 100 μM dithiothreitol (DTT, Sigma), 0.4 μl T4 Polymerase (NEB). DNAse- and RNAse-free water (Gibco, Invitrogen) was used to make up a total volume of 20 μl. This solution was incubated at 22°C for 40 minutes and then at 75°C for 20 minutes to inactivate the T4 polymerase.

### General primer design for cDNA of interest

In order to generate the cDNA fragment to be cloned into the pBLIC backbone, the following PCR primer design was utilized to contain complementary sequences to the T4 exonuclease activity-generated overhangs in pBLIC:

Sense: 5' CACACCATCTCAC**G**-*GCCACC*-the first 20 bases of cDNA starting with ATG

Antisense: 5' CTCACATTCCAC**G**-20 bases from the 3' end of the cDNA

The italicized nucleotides in the forward primer correspond to the Kozak sequence [11], to improve translational efficiency. For primers specific to Bax, catalase and p53, see Table 1.

### PCR amplification of insert and addition of complementary overhangs

Samples for PCR amplification were made by adding 50 ng of DNA, 1 μl of 10 μM dNTP, 5 μL of 10X PCR buffer, 1.5 μl of 50 mM MgCl_2_, 1 μl each of forward and reverse primer at a 10 μM final concentration, 0.5 μl of Platinum taq DNA polymerase (Invitrogen) and dH_2_O for a 50 μl final reaction volume. Gradient PCR (Eppendorf) with annealing temperatures between 55-67°C and 35 cycles was used for optimal primer extension. Unincorporated dNTPs from the PCR samples were removed using a gel purification kit (Qiagen). This was subsequently treated with T4 polymerase (NEB). Treatment conditions were as follows: 0. 2 pmol annealed DNA, 2 μl 10X Buffer 2 (NEB), 2 μl dCTP (Roche), 1 μl 100 μM DTT, 0.4 μl of T4 Polymerase (NEB) and DNAse- and RNAse-free water (Gibco, Invitrogen) to make a total volume of 20 μl. This solution was incubated at 22°C for 40 minutes and then at 75°C for 20 minutes to inactivate T4 polymerase.

### Bacterial transformation and amplification of the pBLIC cDNA retroviral mammalian construct

The T4-treated pBLIC construct (0.04 pmol) and cDNA fragment (0.04 pmol) were mixed together using 1 μl of the former and 2 μl of the latter, and incubated for 5 minutes at 22°C. Instead of a ligation step, 1 μl of 25 mM EDTA was added to a final solution volume of 4 μl for a further 5-minute incubation period to stabilize the non-covalent interactions between the DNA backbone and insert. Approximately 1 μl of this mixture was used to transform recombination-deficient competent XL-10 Gold bacteria (Stratagene) and plated on ampicillin-agar plates (final ampicillin concentration: 100 μg/ml). Competent colonies were selected for inoculation in 100 μg/ml ampicillin-containing LB media, and the resulting DNA plasmid was purified using Qiagen DNA mini and midi prep kits.

### Verification of cloned construct by enzymatic treatment and DNA sequencing

Purified cloned plasmids were analyzed for the appropriate cDNA insert by digesting the constructs with HindIII to detect a shift in size equal to the size of Bax, catalase or p53. Additionally, using sequencing primers against pBABE (pBABE5': CTTTATCCAGCCCTCAC / pBABE3': ACCCTAACTGACACACATTCC), constructs were sequenced at the University of Miami Oncogenomics Facility to verify that the target cDNAs were present in the respective cloned pBLIC construct (Additional File 1, Figure S2).

### Retroviral transduction of constructs

The pBLIC-Bax, pBLIC-catalase and pBLIC-p53 constructs were transduced as described previously [2,12] into the LNCaP human prostate cancer cells or H358 human lung cancer cells. Briefly viral particles were produced by co-transfecting 3 μg MuLV gag-pol-expressing plasmid pUMVC, 300 ng envelope protein-expressing plasmid pCMV.VSV-G and 3-4 μg target construct into HEK 293T cells. The transfection complex was produced in serum-free DMEM media via the transfection agent Fugene^®^6 (Roche) at Fugene (μl): total DNA (μg) ratio of approximately 2:1. The viral supernatants from 293T cells were harvested at 48 hours and 72 hours and applied to the LNCaP cells for 6-8 hours in the presence of 6 μg/ml protamine sulfate. At 48 hours after the last transduction, 500 μg/ml G418 was added to the cell culture media and to a mock-transduced plate of cells. Cells were subsequently continuously selected in G418-containing media to enrich for successfully transduced cells.

### Reverse-transcriptase PCR

Total RNA was isolated using the RNAqueous-4PCR Kit (Ambion) as per manufacturer instructions. A total of 0.25 μg purified RNA was reverse-transcribed using a random primer to obtain cDNA via the High Capacity cDNA Reverse Transcription Kit (Applied Biosystems). The cDNA was used as a template for PCR amplification using AmpliTaq Gold^® ^360 Master Mix (Applied Biosystems) via the primers listed in Table 2. The reactions were run on a 1.5% agarose gel and bands were visualized by ethidium bromide staining. GAPDH was used as an internal loading control.

**Table 2:** RT-PCR primers for detecting cDNA transcript

RT-PCR Primers	Primer Sequence
Bax forward primer	5' CCCGAGAGGTCTTTTTCCGAG 3'
Bax reverse primer	5' CCAGCCCATGATGGTTCTGAT 3'
Catalase forward primer	5' CGCAGAAAGCTGATGTCCTGA 3'
Catalase reverse primer	5' TCATGTGTGACCTCAAAGTAGC 3'
p53 forward primer	5' GAGGTTGGCTCTGACTGTACC 3'
p53 reverse primer	5' TCCGTCCCAGTAGATTACCAC 3'
GAPDH forward primer	5' GACCCCTTCATTGACCTCAAC 3'
GAPDH reverse primer	5' CTTCTCCATGGTGGTGAAGA 3'

### Western Blotting of proteins

Protein lysates were made from harvested cell pellets by resuspending in sodium fluoride (NaF; 50 mM Tris PH 7.5, 150 mM NaCl, 1% Nonidet P-40, 50 mM NaF) lysis buffer (10 μL 0.1 M sodium vanadate (NaVO_3_), 20 μL 50× protease inhibitor, 9 μL of 100 mM phenylmethylsulfonyl fluoride (PMSF), 1 μL of 1 M DTT per 1 ml NaF base buffer). Samples were incubated on ice for 30 min and then centrifuged at 14,000 rpm for 20 min. Protein concentrations were determined using the Bradford assay (5X Bradford Reagent, Biorad). Subsequently 35 μg of protein from each sample was prepared for immunoblotting on a 4-12% Bis-Tris gradient pre-cast gel (Nupage, Invitrogen) on a Novex immunoblotting module (Invitrogen). The gel was run at 120 V for 2 hrs on ice and then was transferred to a PDVF membrane (Immobilon, Millipore) at 35 V for 1.75 hrs in cold room. After transfer, the membrane was immersed in Ponceau reagent (Sigma) to assess relative loading among the various lanes. The membrane was blocked in 5% non-fat dry milk in 0.1% Tween/1X TBS (TBST), incubated with the appropriate antibodies: Bax (1:4000, sc-493, Santa Cruz Biotechnology Inc.), catalase, (1:4000, ab16731, Abcam), p53 (1:1000, sc-126, Santa Cruz Biotechnology Inc.), and GAPDH (1:4000, ab9485, Abcam). Subsequently the blots were washed in 0.1% TBST. After the incubation period in the appropriate horseradish peroxidase-conjugated secondary antibodies (GE Healthcare, Amersham), the blots were again washed in 0.1% TBST. Blots were then exposed to autoradiographic films and developed with the ECLPlus Western Chemiluminescent Detection System (GE Healthcare, Amersham) to determine levels of protein expression.

## Abbreviations

**LIC**: ligation-independent cloning; **MMLV**: Moloney murine leukemia virus; **RT-PCR**: reverse transcriptase polymerase chain reaction.

## Authors' contributions

AP and PR developed the system and designed experiments. AP, AM and KH carried out experiments. PR wrote the manuscript with input from the other authors. All authors have read and approved the final manuscript.

## Supplementary Material

Additional file 1 **Figure S1**. Map of pBLIC-neo. The LIC adaptor is annotated at bases 1361-1386 with the sequencing primers used to verify insertion noted within the diagram. The BamHI and PmlI sites are also noted. The actual sequence around the LIC adaptor site is shown below the map, with key elements highlighted (see Figure 1 for additional details). Figure S2. Sequencing verification of pBLIC.Bax, pBLIC.catalase and pBLIC.p53 constructs. Positive clones (screened by restriction digestion in Figure 3) were sequenced at the Oncogenomics Core facility, University of Miami, FL. The sequences presented here were analyzed using the BLAST algorithm at the NCBI website and found to be accurate. Start and stop codons are highlighted in red text. (A) Bax sequence. Please note that a single A -> G base mismatch (underlined) corresponds to a UCA to UCG silent codon mutation as both sequences encode for serine. (B) Catalase sequences. A small portion consisting of 74 bases was not covered by the catalase primer sets; however the presence of start and stop codons in conjunction with the RT-PCR and Western blot data presented in Figure 4 verify that the full cDNA was successfully cloned within pBLIC-neo. (C) p53 sequences. Click here for file
