# Supplementary material for: Creation and validation of a ligation-independent cloning (LIC) retroviral vector for stable gene transduction in mammalian cells
Source: BMC Biotechnol. 2012 Jan 16;12:3. doi: 10.1186/1472-6750-12-3 (PMC3298557; doi:10.1186/1472-6750-12-3)
Supplement: Additional file 1 — Figure S1. Map of pBLIC-neo. The LIC adaptor is annotated at bases 1361-1386 with the sequencing primers used to verify insertion noted within the diagram. The BamHI and PmlI sites are also noted. The actual sequence around the LIC adaptor site is shown below the map, with key elements highlighted (see Figure 1 for additional details). Figure S2. Sequencing verification of pBLIC.Bax, pBLIC.catalase and pBLIC.p53 constructs. Positive clones (screened by restriction digestion in Figure 3) were sequenced at the Oncogenomics Core facility, University of Miami, FL. The sequences presented here were analyzed using the BLAST algorithm at the NCBI website and found to be accurate. Start and stop codons are highlighted in red text. (A) Bax sequence. Please note that a single A -> G base mismatch (underlined) corresponds to a UCA to UCG silent codon mutation as both sequences encode for serine. (B) Catalase sequences. A small portion consisting of 74 bases was not covered by the catalase primer sets; however the presence of start and stop codons in conjunction with the RT-PCR and Western blot data presented in Figure 4 verify that the full cDNA was successfully cloned within pBLIC-neo. (C) p53 sequences. [file 1472-6750-12-3-S1.PDF]

Supplementary Figure Legends

Figure S1. Map of pBLIC-neo. The LIC adaptor is annotated at bases 1361-1386 with the sequencing primers used to verify insertion noted within the diagram. The BamHI and PmlI sites are also noted. The actual sequence around the LIC adaptor site is shown below the map, with key elements highlighted (see Figure 1 for additional details).

Figure S2. Sequencing verification of pBLIC.Bax, pBLIC.catalase and pBLIC.p53 constructs. Positive clones (screened by restriction digestion in Figure 3) were sequenced at the Oncogenomics Core facility, University of Miami, FL. The sequences presented here were analyzed using the BLAST algorithm at the NCBI website and found to be accurate. Start and stop codons are highlighted in red text. (A) Bax sequence. Please note that a single A -> G base mismatch (underlined) corresponds to a UCA to UCG silent codon mutation as both sequences encode for serine. (B) Catalase sequences. A small portion consisting of 74 bases was not covered by the catalase primer sets; however the presence of start and stop codons in conjunction with the RT-PCR and Western blot data presented in Figure 4 verify that the full cDNA was successfully cloned within pBLIC-neo. (C) p53 sequences.

Figure S1

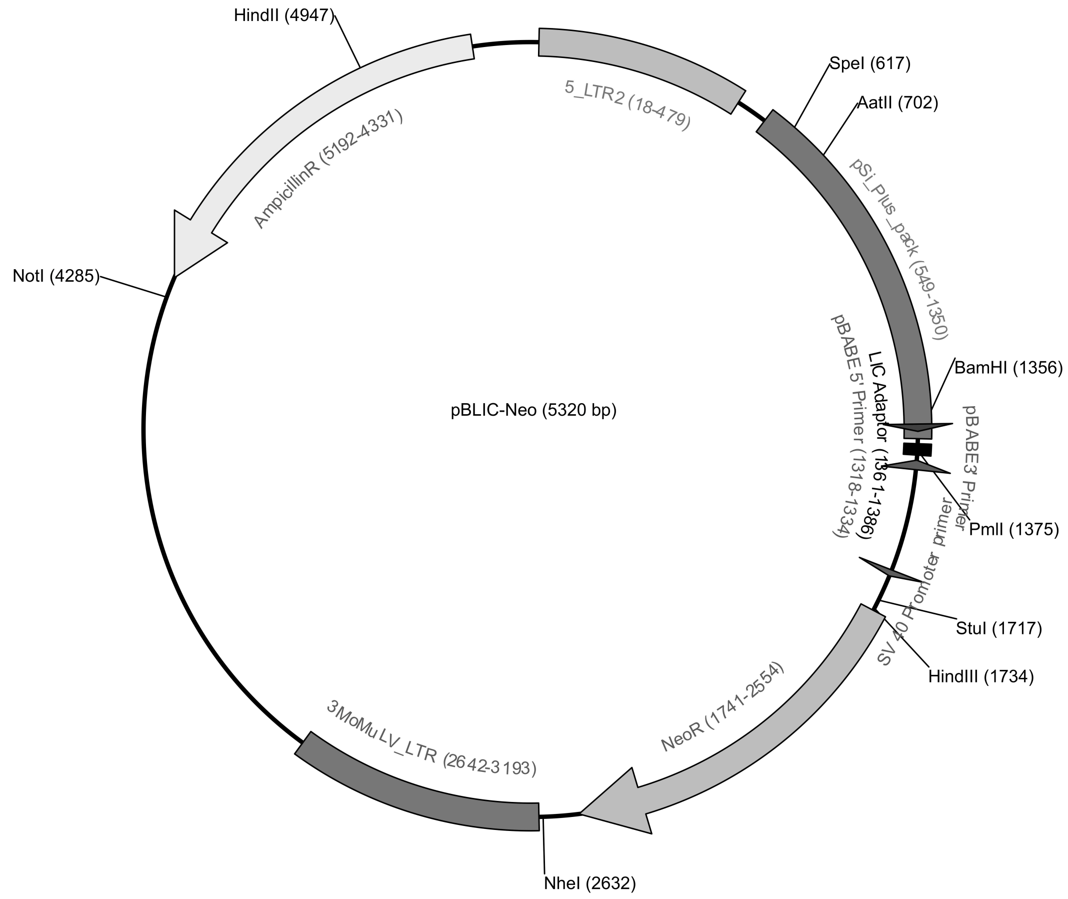

CCTTCTCTAGGCGCCGGCCGGATCCGCACACCATCTCACGTGGAATGTGAGCTCGACCCTGTGGAATGTGTGTCAG
GGAAGAGATCCGCGGCCGGCCTAGGCGTGTGGTAGAGTGCACCTTACACTCGAGCTGGGACACCTTACACACAGTC

Red- BamHI site (1356)
Blue bold- G, termination point for T4 activity
Green bold – PmlI site (1375)

Figure S2

A.

Bax sequence 1

TCTCACGGCCACCATGGACGGGTCCGGGGAGCAGCCCAGAGGCGGGGGCCCACCAGCTCTGAGCAGATCATGAAGACAGGG
GCCCTTTTGCTTCAGGGTTTCATCCAGGATCGAGCAGGGCGAATGGGGGGGGAGGCACCCGAGCTGGCCCTGGACCCGGTGCC
TCAGGATGCGTCCACCAAGAAGCTGAGCGAGTGTCTCAAGCGCATCGGGGACGAACTGGACAGTAACATGGAGCTGCAGAGG
ATGATTGCCGCCGTGGACACAGACTCCCCCCGAGAGGTCTTTTTCCGAGTGGCAGCTGACATGTTTTCTGACGGCAACTTCAAC
TGGGGCCGGGTTGTCGCCCTTTTCTACTTTGCCAGCAAACTGGTGCTCAAGGCCCTGTGCACCAAGGTGCCGGAACTGATCAGA
ACCATCATGGGCTGGACATTGGACTTCCTCCGGGAGCGGCTGTTGGGCTGGATCCAAGACCAGGGTGGTTGGGACGGCCTCCTC
TCCTACTTTGGGACGCCCACGTGGCAGACCGTGACCATCTTTGTGGCGGGAGTGCTCACCGCCTCGCTCACCATCTGGAAGAAG
ATGGGCTGACGTGGAATGTGAGCTCGACCCTGTGGAATGTGTGTCAGTTAGGGTGTGGAAAGTCCCCAGGCTCCCCAGCAGGC
AGAAGTATGCAAAGCATGCATCTCAATTAGTCAGCAACCAGGTGTGGAAAGTCCCCAGGCTCCCCAGCAGGCAGAAGTATGCA
AAGCATGCATCTCAATTAGTCAGCAACCATAGTCCCGCCCCTAACTCCGCCCATCCCGCCCCTAACTCCGCCCAGT

Figure S2

B.

Catalase sequence 1

CCATCTCACGGCCACCATGGCTGACAGCCGGGATCCCGCCAGCGACCAGATGCAGCACTGGAAGGAGCAGCGGGCCGC
GCAGAAAGCTGATGTCCTGACCACTGGAGCTGGTAACCCAGTAGGAGACAAACTTAATGTTATTACAGTAGGGCCCCGT
GGGCCCCTTCTTGTTCAGGATGTGGTTTTCACTGATGAAATGGCTCATTTTGACCGAGAGAGAATTCCTGAGAGAGTTGT
GCATGCTAAAGGAGCAGGGGCCTTTGGCTACTTTGAGGTCACACATGACATTACCAAATACTCCAAGGCAAAGGTATTTG
AGCATATTGGAAAGAAGACTCCCATCGCAGTTCGGTTCTCCACTGTTGCTGGAGAATCGGGTTCAGCTGACACAGTTCG
GGACCCTCGTGGGTTTGCAGTGAAATTTACACAGAAGATGGTAACTGGGATCTCGTTGGAAATAACACCCCCATTTTCT
TCATCAGGGATCCCATATTGTTTCCATCTTTTATCCACAGCCAAAAGAGAAATCCTCAGACACATCTGAAGGATCCGGACA
TGGTCTGGGACTTCTGGAGCCTACGTCCTGAGTCTCTGCATCAGGTTTCTTTCTTGTTCAGTGATCGGGGGATTCCAGATG
GACATCGCCACATGAATGGATATGGATCACATACTTTCAAGCTGGTTAATGCAAATGGGGGAGGCAGTTTATTGCAAATTC
CATTATAAGACTGACCAGGGCATCAAAAACCTTTCTGTTGAAGATGCGGCGAGACTTTCCCAGGAAGATCCTGACTATGG
GCATCCGGGGATCTTTTTAACGCCATT

Catalase sequence 2

TCGTTCGAACCCCGCCTCGATCCTCCCTTTATCCCAGCCCTCACTCCTTCTCTAGGCGCCGGCCGGATCCGCACACCATCT
CACGGCCACCATGGCTGACAGCCGGGATCCCGCCAGCGACCAGATGCAGCACTGGAAGGAGCAGCGGGCCGCGCAGA
AAGCTGATGTCCTGACCACTGGAGCTGGTAACCCAGTAGGAGACAAACTTAATGTTATTACAGTAGGGCCCCGTGGGCC
CCTTCTTGTTCAGGATGTGGTTTTCACTGATGAAATGGCTCATTTTGACCGAGAGAGAATTCCTGAGAGAGTTGTGCATG
CTAAAGGAGCAGGGGCCTTTGGCTACTTTGAGGTCACACATGACATTACCAAATACTCCAAGGCAAAGGTATTTGAGCAT
ATTGGAAAGAAGACTCCCATCGCAGTTCGGTTCTCCACTGTTGCTGGAGAATCGGGTTCAGCTGACACAGTTCGGGACC
CTCGTGGGTTTGCAGTGAAATTTACACAGAAGATGGTAACTGGGATCTCGTTGGAAATAACACCCCCATTTTCTTCATCA
GGGATCCCATATTGTTTCCATCTTTTATCCACAGCCAAAAGAGAAATCCTCAGACACATCTGAAGGATCCGGACATGGTCT
GGGACTTCTGGAGCCTACGTCCTGAGTCTCTGCATCAGGTTTCTTTCTTGTTCAGTGATCGGGGGATTCCAGATGGACATC
GCCACATGAATGGATATGGATCACATACTTTCAAGCTGGTTAATGCAAATGGGGGAGGCAGTTTATTGCAAATTCCATTATA
AGACTGACCAGGGCATCAAAAACCTTTCTGTTGAAGATGCGGCGAGACTTCCCAGGAAGAT

Catalase sequence 3

TTAATCAGGCAGAAACTTTCCATTAATCCATTTGATCTCACCAAGGTTTGGCCTCACAAGGACTACCCTCTCATCCCAGTT
GGTAAACTGGTCTTAAACCGGAATCCAGTTAATTACTTTGCTGAGGTTGAACAGATAGCCTTCGACCCAAGCAACATGCC
ACCTGGCATTGAGGCCAGTCCTGACAAAATGCTTCAGGGCCGCCTTTTTGCCTATCCTGACACTCACCGCCATCGCCTGG
GACCCAATTATCTTCATATACCTGTGAACTGTCCCTACCGTGCTCGAGTGGCCAACTACCAGCGTGACGGCCCGATGTGC
ATGCAGGACAATCAGGGTGGTGCTCCAAATTACTACCCCAACAGCTTTGGTGCTCCGGAACAACAGCCTTCTGCCCTGG
AGCACAGCATCCAATATTCTGGAGAAGTGCGGAGATTCAACACTGCCAATGATGATAACGTTACTCAGGTGCGGGCATTC
TATGTGAACGTGCTGAATGAGGAACAGAGGAAACGTCTGTGTGAGAACATTGCCGGCCACCTGAAGGATGCACAAATTT
TCATCCAGAAGAAAGCGGTCAAGAACTTCACTGAGGTCCACCCTGACTACGGGAGCCACATCCAGGCTCTTCTGGACAA
GTACAATGCTGAGAAGCCTAAGAATGCGATTCACACCTTTGTGCAGTCCGGATCTCACTTGGCGGCAAGGGAGAAGGCA
AATCTGTGAGCC

Figure S2

C.

p53 sequence 1

CCATCTCACGGCCACCATGGAGGAGCCGCAGTCAGATCCTAGCGTCGAGCCCCCTCTGAGTCA
GGAAACATTTTCAGACCTATGGAAACTACTTCCTGAAAACAACGTTCTGTCCCCCTTGCCGTC
CCAAGCAATGGATGATTTGATGCTGTCCCCGGACGATATTGAACAATGGTTCACTGAAGACCC
AGGTCCAGATGAAGCTCCCAGAATGCCAGAGGCTGCTCCCCCCGTGGCCCCTGCACCAGCAG
CTCCTACACCGGCGGCCCCTGCACCAGCCCCCTCCTGGCCCCTGTCATCTTCTGTCCCTTCCCA
GAAAACCTACCAGGGCAGCTACGGTTTCCGTCTGGGCTTCTTGCATTCTGGGACAGCCAAGTC
TGTGACTTGCACGTACTCCCCTGCCCTCAACAAGATGTTTTGCCAACTGGCCAAGACCTGCCCT
GTGCAGCTGTGGGTTGATTCCACACCCCCGCCCGGCACCCGCGTCCGCGCCATGGCCATCTAC
AAGCAGTCACAGCACATGACGGAGGTTGTGAGGCGCTGCCCCCACCATGAGCGCTGCTCAGA
TAGCGATGGTCTGGCCCCTCCTCAGCATCTTATCCGAGTGGAAGGAAATTTGCGTGTGGAGTA
TTTGGATGACAGAAACACTTTTCGACATAGTGTGGTGGTGCCCTATGAGCCGCCTGAGGTTGG
CTCTGACTGTACCACCATCCACTACAACTACATGTGTAACAGTTCCTGCATGGGCGGCATGAA
CCGGAGGCCCATCCTCACCATCATCACACTGGAAGACTCCAGTGGTAATCTACTGGGA

p53 sequence 2

TCTGGGCTTCTTGCATTCTGGGACAGCCAAGTCTGTGACTTGCACGTACTCCCCTGCCCTCAAC
AAGATGTTTTGCCAACTGGCCAAGACCTGCCCTGTGCAGCTGTGGGTTGATTCCACACCCCCC
GCCCGGCACCCGCGTCCGCGCCATGGCCATCTACAAGCAGTCACAGCACATGACGGAGGTTG
TGAGGCGCTGCCCCCACCATGAGCGCTGCTCAGATAGCGATGGTCTGGCCCCTCCTCAGCATC
TTATCCGAGTGGAAGGAAATTTGCGTGTGGAGTATTTGGATGACAGAAACACTTTTCGACATA
GTGTGGTGGTGCCCTATGAGCCGCCTGAGGTTGGCTCTGACTGTACCACCATCCACTACAACT
ACATGTGTAACAGTTCCTGCATGGGCGGCATGAACCGGAGGCCCATCCTCACCATCATCACAC
TGGAAGACTCCAGTGGTAATCTACTGGGACGGAACAGCTTTGAGGTGCGTGTTTGTGCCTGTC
CTGGGAGAGACCGGCGCACAGAGGAAGAGAATCTCCGCAAGAAAGGGGAGCCTCACCACGA
GCTGCCCCCAGGGAGCACTAAGCGAGCACTGCCCAACAACACCAGCTCCTCTCCCCAGCCAA
AGAAGAAACCACTGGATGGAGAATATTTCACCCTTCAGATCCGTGGGCGTGAGCGCTTCGAG
ATGTTCCGAGAGCTGAATGAGGCCTTGGAACTCAAGGATGCCCAGGCTGGGAAGGAGCCAGG
GGGGAGCAGGGCTCACTCCAGCCACCTGAAGTCCAAAAAGGGTCAGTCTACCTCCCGCCATA
AAAAACTCATGTTCAAGACAGAAGGGCCTGACTCAGACTGACGTG
